# Algorithm for finding partitionings of hard variants of boolean satisfiability problem with application to inversion of some cryptographic functions

**DOI:** 10.1186/s40064-016-2187-4

**Published:** 2016-04-30

**Authors:** Alexander Semenov, Oleg Zaikin

**Affiliations:** Laboratory of Discrete Analysis and Applied Logic, Matrosov Institute for System Dynamics and Control Theory of Siberian Branch of Russian Academy of Sciences, 292, Lermontov Str., 134, Irkutsk, 664033 Russia

**Keywords:** Boolean satisfiability problem, SAT, SAT-based cryptanalysis, Partitioning, Monte Carlo method, Simulated annealing, Tabu search, SAT@home

## Abstract

In this paper we propose an approach for constructing partitionings of hard variants of the Boolean satisfiability problem (SAT). Such partitionings can be used for solving corresponding SAT instances in parallel. For the same SAT instance one can construct different partitionings, each of them is a set of simplified versions of the original SAT instance. The effectiveness of an arbitrary partitioning is determined by the total time of solving of all SAT instances from it. We suggest the approach, based on the Monte Carlo method, for estimating time of processing of an arbitrary partitioning. With each partitioning we associate a point in the special finite search space. The estimation of effectiveness of the particular partitioning is the value of predictive function in the corresponding point of this space. The problem of search for an effective partitioning can be formulated as a problem of optimization of the predictive function. We use metaheuristic algorithms (simulated annealing and tabu search) to move from point to point in the search space. In our computational experiments we found partitionings for SAT instances encoding problems of inversion of some cryptographic functions. Several of these SAT instances with realistic predicted solving time were successfully solved on a computing cluster and in the volunteer computing project SAT@home. The solving time agrees well with estimations obtained by the proposed method.

## Background

The Boolean satisfiability problem (SAT) consists in the following: for an arbitrary Boolean formula to decide if it is satisfiable, i.e. if there exists such an assignment of Boolean variables from the formula that makes this formula true. SAT is usually considered for a Boolean formula in conjunctive normal form (CNF), because SAT for any Boolean formula can be effectively reduced to SAT for some CNF. Despite the fact that SAT is an NP-hard problem, it has wide spectrum of practical applications because many combinatorial problems from different areas can be reduced to it (Biere et al. 2009). The effectiveness of the SAT solving algorithms in the recent years dramatically increased. At the present moment these algorithms are often used in formal verification, combinatorics, cryptanalysis, bioinformatics and other areas.

In this paper we consider the applicability of the SAT approach to problems of inversion of some cryptographic functions. The corresponding SAT instances are hard and the success in their solving has at least two positive consequences. First, the SAT solving algorithms, that successfully cope with cryptanalysis instances, are powerful computing methods and can be applied to solving combinatorial problems from various classes. Second, they can be used to justify the resistance of cryptographic systems or to find their vulnerabilities. Unfortunately, today there is no unified term to represent the cryptanalysis via SAT approach. In the corresponding papers such phrases as “logical cryptanalysis”, “SAT-aided cryptanalysis”, “cryptanalysis via SAT solvers”, etc. are used for this purpose. Hereinafter we will refer to it as *SAT-based cryptanalysis*.

The development of parallel SAT solving algorithms is very relevant. There are two approaches to constructing such algorithms: the portfolio approach and the partitioning approach. According to the portfolio approach one SAT instance is solved using different SAT solvers. During their work, SAT solvers share information (usually in the form of conflict clauses). According to the partitioning approach the original SAT instance is decomposed into a family of independent instances. For solving instances from the obtained family it is natural to use a parallel or a distributed computing system. Note that for a particular SAT instance there can be constructed a lot of different partitionings. In this case there arises a question: how to evaluate a partitioning and compare it to others? From the practical point of view it can be reformulated as follows: how to find relatively good partitioning in a reasonable time? In this paper we answer these questions.

Below we present the brief outline of this paper. First, we consider the problem of estimating the effectiveness of a SAT partitioning as a problem of estimating the expected value of a special random variable. To solve the latter problem we apply the Monte Carlo method in its classical formulation (Metropolis and Ulam 1949). Then the problem of finding a SAT partitioning with a realistic estimated time, required to process it, is reduced to the optimization problem for the predictive function in a special finite search space. We use two metaheuristic algorithms to solve this problem: simulated annealing and tabu search.

The proposed methods for constructing SAT partitionings were tested in application to cryptanalysis of two well known stream ciphers: A5/1 and Bivium. The search for SAT partitionings was performed using a computing cluster. The corresponding cryptanalysis instances were solved using the found partitionings on the computing cluster and also in the volunteer computing project SAT@home, that was developed by us specifically for the purpose of solving hard SAT instances via partitioning approach.

We would like to emphasize that this paper is a significant extension of the paper (Semenov and Zaikin 2015), which appeared in the proceedings of 13th international conference on parallel computing technologies (PaCT’2015).

## Monte Carlo approach to statistical estimation of effectiveness of SAT partitioning

Let us consider the SAT for an arbitrary CNF *C*. The partitioning of *C* is a set of formulas

$$\begin{aligned} C\wedge G_j,\quad j\in \{1,\ldots ,s\} \end{aligned}$$

such that for any $i,j:i\ne j$ formula $C \wedge G_i \wedge G_j$ is unsatisfiable and

$$\begin{aligned} C\equiv C \wedge G_1 \vee \cdots \vee C \wedge G_s. \end{aligned}$$

(hereinafter by “$\equiv$” we denote logical equivalence). Obviously when one has a partitioning of the original SAT instance, SAT for formulas $C \wedge G_j$, $j\in \{1,\ldots ,s\}$ can be solved independently in parallel.

There exist various partitioning techniques. For example one can construct $\{G_j \}_{j=1}^s$ using a scattering procedure, a guiding path solver, lookahead solver and a number of other techniques described in Hyvärinen (2011). Unfortunately, for these partitioning methods it is hard in general case to estimate the time required to solve an original problem. From the other hand in a number of papers about SAT-based cryptanalysis of several keystream ciphers there was used a partitioning method that makes it possible to construct such estimations in quite a natural way. In particular, in Eibach et al. (2008), Soos et al. (2009), Soos (2010), Zaikin and Semenov (2008) for this purpose the information about the time to solve small number of subproblems randomly chosen from the partitioning of an original problem was used. In our paper we give strict formal description of this idea within the borders of the Monte Carlo method in its classical form (Metropolis and Ulam 1949). Also we focus our attention on some important details of the method that were not considered in previous works.

Consider SAT for an arbitrary CNF *C* over a set of Boolean variables $X=\{x_1,\ldots ,x_n\}$. To an arbitrary set $\tilde{X}=\left\{ x_{i_1},\ldots ,x_{i_d}\right\}$, $\tilde{X}\subseteq X$ we refer as a decomposition set. Consider a partitioning of *C* that consists of a set of $2^d$ formulas

$$\begin{aligned} C \wedge G_j,\quad j\in \{1,\ldots ,2^d\} \end{aligned}$$

where $G_j$, $j\in \{1,\ldots ,2^d\}$ are all possible minterms over $\tilde{X}$. Note that an arbitrary formula $G_j$ takes a value of true on a single truth assignment $\left( \alpha _1^j,\ldots ,\alpha _d^j\right) \in \{0,1\}^d$. Therefore, an arbitrary formula $C \wedge G_j$ is satisfiable if and only if $C\left[ \tilde{X} /\left( \alpha _1^j,\ldots ,\alpha _d^j\right) \right]$ is satisfiable. Here $C\left[ \tilde{X} /\left( \alpha _1^j,\ldots ,\alpha _d^j\right) \right]$ is produced by setting values of variables $x_{i_k}$ to corresponding $\alpha _k^j$, $k\in \{1,\ldots ,d\}$ : $x_{i_1}=\alpha _1^j,\ldots ,x_{i_d}=\alpha _d^j$. To a set of CNFs

$$\begin{aligned} \Delta _C(\tilde{X})=\left\{ C\left[ \tilde{X}/\left( \alpha _1^j,\ldots ,\alpha _d^j\right) \right] \right\} _{\left( \alpha _1^j,\ldots ,\alpha _d^j\right) \in \{0,1\}^d} \end{aligned}$$

we will refer as a decomposition family produced by $\tilde{X}$. It is easy to see that the decomposition family is the partitioning of the SAT instance *C*.

Let *A* be some SAT solving algorithm. Hereinafter we presume that *A* is complete, i.e. it halts on every input. We also presume that *A* is a non-randomized deterministic algorithm. We denote the total runtime of *A* on all the SAT instances from $\Delta _C\left( \tilde{X}\right)$ as $t_{C,A}\left( \tilde{X}\right)$. Below we suggest a method for estimating $t_{C,A}\left( \tilde{X}\right)$.

Define the uniform distribution on the set $\{0,1\}^d$. With each randomly chosen truth assignment $\left( \alpha _1,\ldots ,\alpha _d\right)$ from $\{0,1\}^d$ we associate a value $\xi _{C,A}\left( \alpha _1,\ldots ,\alpha _d\right)$ that is equal to the runtime of *A* on CNF $C\left[ \tilde{X} /\left( \alpha _1,\ldots ,\alpha _d\right) \right]$. Let $\xi ^1,\ldots ,\xi ^Q$ be all the different values that $\xi _{C,A}\left( \alpha _1,\ldots ,\alpha _d\right)$ takes on all the possible $\left( \alpha _1,\ldots ,\alpha _d\right) \in \{0,1\}^d$. Below we use the following notation

1 $$\begin{aligned} \xi _{C,A}\left( \tilde{X}\right) =\left\{ \xi ^1,\ldots ,\xi ^Q\right\} . \end{aligned}$$

Denote the number of $\left( \alpha _1,\ldots ,\alpha _d\right)$, such that $\xi _{C,A}\left( \alpha _1,\ldots ,\alpha _d\right) =\xi ^j$, as $\sharp \xi ^j$. Associate with (1) the following set

$$\begin{aligned} P\left( \xi _{C,A}\left( \tilde{X}\right) \right) =\left\{ \frac{\sharp \xi ^1}{2^d},\ldots ,\frac{\sharp \xi ^Q}{2^d}\right\} . \end{aligned}$$

We say that the random variable $\xi _{C,A}\left( \tilde{X}\right)$ has distribution $P\left( \xi _{C,A}\left( \tilde{X}\right) \right)$. Note that the following equality holds

$$\begin{aligned} t_{C,A}\left( \tilde{X}\right) =\sum \limits _{k=1}^Q \left( \xi ^k\cdot \sharp \xi ^k\right) =2^d\cdot \sum \limits _{k=1}^Q\left( \xi ^k\cdot \frac{\sharp \xi ^k}{2^d}\right) . \end{aligned}$$

Therefore,

2 $$\begin{aligned} t_{C,A}\left( \tilde{X}\right) =2^d\cdot \mathrm {E}\left[ \xi _{C,A}\left( \tilde{X}\right) \right] . \end{aligned}$$

To estimate the expected value $\mathrm {E}\left[ \xi _{C,A}\left( \tilde{X}\right) \right]$ we will use the Monte Carlo method (Metropolis and Ulam 1949). According to this method, a probabilistic experiment that consists of *N* independent observations of values of an arbitrary random variable $\xi$ is used to approximately calculate $\mathrm {E}\left[ \xi \right]$. Let $\zeta ^1,\ldots ,\zeta ^N$ be results of the corresponding observations. They can be considered as a single observation of *N* independent random variables with the same distribution as $\xi$. If $\mathrm {E}\left[ \xi \right]$ and $\mathrm {Var}\left( \xi \right)$ are both finite then from the Central Limit Theorem (Feller 1971) we have the main formula of the Monte Carlo method

3 $$\begin{aligned} \mathrm {Pr}\left\{ \left| \frac{1}{N}\cdot \sum \limits _{j=1}^N \zeta ^j - \mathrm {E}\left[ \xi \right] \right| <\frac{\delta _\gamma \cdot \sigma }{\sqrt{N}}\right\} =\gamma . \end{aligned}$$

Here $\sigma =\sqrt{Var\left( \xi \right) }$ stands for a standard deviation, $\gamma$ – for a confidence level, $\gamma =\Phi \left( \delta _\gamma \right)$, where $\Phi \left( \cdot \right)$ is the normal cumulative distribution function. It means that under the considered assumptions the value

$$\begin{aligned} \frac{1}{N}\cdot \sum \limits _{j=1}^N \zeta ^j \end{aligned}$$

is a good approximation of $\mathrm {E}\left[ \xi \right]$, when the number of observations *N* is large enough.

Due to completeness of *A* the expected value and variance of random variable $\xi _{C,A}(\tilde{X})$ are finite. Since *A* is deterministic (i.e. it does not use randomization) the observed values will have the same distribution. One can use the preprocessing stage to estimate the effectiveness of the considered partitioning because *N* can be significantly less than $2^d$.

So the process of estimating the value (2) for a given $\tilde{X}$ is as follows. We randomly choose *N* truth assignments of variables from $\tilde{X}$

4 $$\begin{aligned} \alpha ^1=\left( \alpha _1^1,\ldots ,\alpha _d^1\right) ,\ldots ,\alpha ^N=\left( \alpha _1^N,\ldots ,\alpha _d^N\right) . \end{aligned}$$

Below we refer to (4) as *random sample*. Then consider values

$$\begin{aligned} \zeta ^j=\xi _{C,A}\left( \alpha ^j\right) ,\quad j=1,\ldots ,N \end{aligned}$$

and calculate the value

5 $$\begin{aligned} F_{C,A}\left( \tilde{X}\right) =2^d\cdot \left( \frac{1}{N}\cdot \sum \limits _{j=1}^N \zeta ^j\right) . \end{aligned}$$

If *N* is large enough then the value of $F_{C,A}\left( \tilde{X}\right)$ can be considered as a good approximation of (2). That is why one can search for a decomposition set with minimal value of $F_{C,A}\left( \cdot \right)$ instead of finding a decomposition set with minimal value (2). Below we refer to function $F_{C,A}\left( \cdot \right)$ as *predictive function*.

## Algorithms for minimization of predictive function

Below we will describe the algorithm for finding good partitionings. This algorithm is based on the procedure minimizing the predictive function in the special search space.

Let *C* be an arbitrary CNF over the set of Boolean variables $X=\left\{ x_1,\ldots ,x_n\right\}$. Let $\tilde{X}\subseteq X$ be an arbitrary decomposition set. We can represent $\tilde{X}$ by binary vector $\chi =\left( \chi _1,\ldots ,\chi _n\right)$. Here

$$\begin{aligned} \chi _i=\left\{ \begin{array}{ll} 1,&{}\quad if\;x_i\in \tilde{X}\\ 0,&{}\quad if\;x_i\notin \tilde{X} \end{array} \right., \quad i\in \{1,\ldots ,n\} \end{aligned}$$

For an arbitrary $\chi \in \{0,1\}^n$ we compute the value of function $F(\chi )$ in the following way. For vector $\chi$ we construct the corresponding set $\tilde{X}$ (it is formed by variables from *X* that correspond to 1 positions in $\chi$). Then we construct a random sample $\alpha ^1,\ldots ,\alpha ^N$, $\alpha ^j\in \{0,1\}^{|\tilde{X}|}$ [see (4)] and solve SAT for CNFs $C\left[ \tilde{X}/\alpha ^j\right]$. For each of these SAT instances we measure $\zeta ^j$ — the runtime of algorithm *A* on the input $C\left[ \tilde{X}/\alpha ^j\right]$. After this we calculate the value of $F_{C,A}\left( \tilde{X}\right)$ according to (5). As a result we have the value of $F(\chi )$ in the considered point of the search space.

Now we will solve the problem $F(\chi )\rightarrow min$ over the set $\{0,1\}^n$. Of course, the problem of search for the exact minimum of function $F(\chi )$ is extraordinarily complex. Therefore our main goal is to find in affordable time the points in $\{0,1\}^n$ with relatively good values of function $F(\cdot )$. Note that the function $F(\cdot )$ is not specified by some formula and therefore we do not know any of its analytical properties. That is why to minimize this function we use metaheuristic algorithms: simulated annealing and tabu search.

First, we need to introduce the notation. By $\mathfrak {R}$ we denote the search space, for example, $\mathfrak {R}=\{0,1\}^n$, however, as we will see later, for the problems considered one can use the search spaces of much less power. During the minimization of function $F(\cdot )$ we iteratively move from one point of the search space to another:

$$\begin{aligned} \chi ^0\rightarrow \chi ^1\rightarrow \cdots \rightarrow \chi ^i\rightarrow \cdots \rightarrow \chi ^{*}. \end{aligned}$$

By $N_{\rho }\left( \chi \right)$ we denote the neighborhood of point $\chi$ of radius $\rho$ in the search space $\mathfrak {R}$. The point from which the search starts we denote as $\chi _{start}$. We will refer to the decomposition set specified by this point as $\tilde{X}_{start}$. The current Best Known Value of $F(\cdot )$ is denoted by $F_{best}$. The point in which the $F_{best}$ was achieved we denote as $\chi _{best}$. By $\chi _{center}$ we denote the point the neighborhood of which is processed at the current moment. We call the point, in which we computed the value $F(\cdot )$, a *checked point*. The neighborhood $N_{\rho }\left( \chi \right)$ in which all the points are checked is called *checked neighborhood*. Otherwise the neighborhood is called *unchecked*.

According to the scheme of the simulated annealing (Kirkpatrick et al. 1983), the transition from $\chi ^i$ to $\chi ^{i+1}$ is performed in two stages. First we choose a point $\tilde{\chi }^i$ from $N_{\rho }\left( \chi ^i\right)$. The point $\tilde{\chi }^i$ becomes the point $\chi ^{i+1}$ with the probability denoted as $\mathrm {Pr}\left\{ \tilde{\chi }^i\rightarrow \chi ^{i+1}|\chi ^i\right\}$. This probability is defined in the following way:

$$\begin{aligned} \mathrm {Pr}\left\{ \tilde{\chi }^i\rightarrow \chi ^{i+1}|\chi ^i\right\} =\left\{ \begin{array}{ll} 1,&{}\quad if\;F\left( \tilde{\chi }^i\right) <F\left( \chi ^i\right) \\ \exp \left( -\frac{F\left( \tilde{\chi }^i\right) -F\left( \chi ^i\right) }{T_i}\right) ,&{} \quad if\;F\left( \tilde{\chi }^i\right) \ge F\left( \chi ^i\right) \end{array} \right. \end{aligned}$$

In the pseudocode of the algorithm demonstrated below, the function that tests if the point $\tilde{\chi }^i$ becomes $\chi ^{i+1}$, is called PointAccepted (this function returns the value of true if the transition occurs and false otherwise). The change of parameter $T_i$ corresponds to decreasing the “temperature of the environment” (Kirkpatrick et al. 1983) (in the pseudocode by decreaseTemperature() we denote the function which implements this procedure). Usually it is assumed that $T_i=Q\cdot T_{i-1}$, $i\ge 1$, where $Q\in (0,1)$. The process starts at some initial value $T_0$ and continues until the temperature drops below some threshold value $T_{inf}$ (in the pseudocode the function that checks this condition is called temperatureLimitReached()).

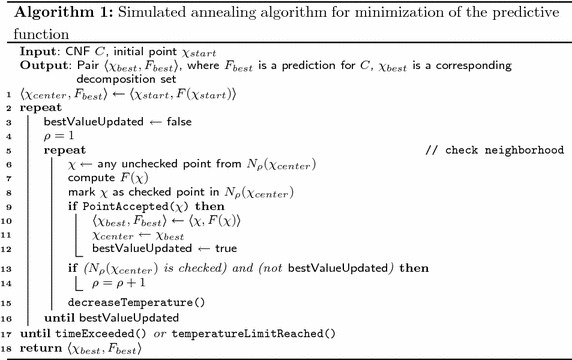



Another metaheuristic scheme that we used for minimization of $F(\cdot )$ is the tabu search algorithm (Glover and Laguna 1997). According to this algorithm we store the points from the search space, in which we already calculated the values of function $F(\cdot )$, in special tabu lists. When we try to improve the current Best Known Value of $F(\cdot )$ in the neighborhood of some point $\chi _{center}$ then for an arbitrary point $\chi$ from the neighborhood we first check if we haven’t computed $F(\chi )$ earlier. If we haven’t and, therefore, the point $\chi$ is not contained in tabu lists, then we compute $F(\chi )$. This strategy is justified in the case of the minimization of predictive function $F(\cdot )$ because the computing of values of the function in some points of the search space can be very expensive. The use of tabu lists makes it possible to significantly increase the number of points of the search space processed per time unit.

Let us describe the tabu search algorithm for minimization $F(\cdot )$ in more detail. To store the information about points, in which we already computed the value of $F(\cdot )$ we use two tabu lists $L_1$ and $L_2$. The $L_1$ list contains only points with checked neighborhoods. The $L_2$ list contains checked points with unchecked neighborhoods. Below we present the pseudocode of the tabu search algorithm for $F(\cdot )$ minimization.

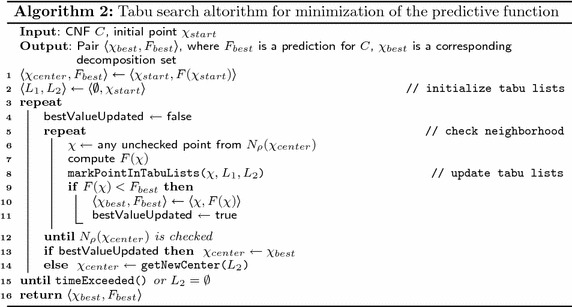



In this algorithm the function markPointInTabuLists$\left( \chi ,L_1,L_2\right)$ adds the point $\chi$ to $L_2$ and then marks $\chi$ as checked in all neighborhoods of points from $L_2$ that contain $\chi$. If as a result the neighborhood of some point $\chi '$ becomes checked, the point $\chi '$ is removed from $L_2$ and is added to $L_1$. If we have processed all the points in the neighborhood of $\chi _{center}$ but could not improve the $F_{best}$ then as the new point $\chi _{center}$ we choose some point from $L_2$. It is done via the function getNewCenter($L_2$). To choose the new point in this case one can use various heuristics. In our current implementation the tabu search algorithm chooses the point for which the total conflict activity (Marques-Silva et al. 2009) of Boolean variables, contained in the corresponding decomposition set, is the largest.

As we already mentioned above, taking into account the features of the considered SAT problems makes it possible to significantly decrease the size of the search space. For example, knowing the so called Backdoor Sets (Williams et al. 2003) can help in that matter. Let us consider the SAT instance that encodes the inversion problem of the function of the kind $f:\{0,1\}^k\rightarrow \{0,1\}^l$. Let *S*(*f*) be the Boolean circuit implementing *f*. Then the set $\tilde{X}_{in}$, formed by the variables encoding the inputs of the Boolean circuit *S*(*f*), is the so called Strong Unit Propagation Backdoor Set (Järvisalo and Junttila 2009). It means that if we use $\tilde{X}_{in}$ as the decomposition set, then the CDCL (Conflict-Driven Clause Learning Marques-Silva et al. 2009) solver will solve SAT for any CNF of the kind $C\left[ \tilde{X}_{in}/\alpha \right]$, $\alpha \in \{0,1\}^{|\tilde{X}_{in}|}$ on the preprocessing stage, i.e. very fast. Therefore the set $\tilde{X}_{in}$ can be used as the set $\tilde{X}_{start}$ in the predictive function minimization procedure. Moreover, in this case it is possible to use the set $2^{\tilde{X}_{in}}$ in the role of the search space $\mathfrak {R}$. In all our computational experiments we followed this path.

## Computational experiments

We implemented the algorithms from the previous section in the form of PDSAT MPI-program (https://github.com/Nauchnik/pdsat). One process of PDSAT is the leader process, all the other are computing processes (each process corresponds to 1 CPU core).

The leader process selects points of the search space (we use neighborhoods of radius $\rho =1$). For every new point $\chi =\chi \left( \tilde{X}\right)$ it generates a random sample (4) of size *N*. Each assignment from (4) combined with the original CNF *C* defines the SAT instance from the decomposition family $\Delta _C\left( \tilde{X}\right)$. These instances are solved by computing processes. When computing the value of the predictive function we assume that the decomposition family will be processed by 1 CPU core. We can extrapolate the estimation obtained to an arbitrary parallel (or distributed) computing system because the processing of $\Delta _C\left( \tilde{X}\right)$ consists in solving independent subproblems. In the computing processes MiniSat solver (Eén and Sörensson 2003) is used. This solver was modified to be able to stop computations upon receiving corresponding messages from the leader process.

Below we present the estimations produced by PDSAT for SAT-based cryptanalysis of the A5/1 (Biryukov et al. 2000), Bivium (Cannière 2006) and Grain (Hell et al. 2007) keystream generators. We used the Transalg system (Otpuschennikov et al. 2015) to construct SAT instances for these problems.

### Time estimations for SAT-based cryptanalysis of A5/1

For the first time the SAT-based cryptanalysis of the A5/1 keystream generator was considered in Semenov et al. (2011). Further we study this problem in the following form: to find the secret key of length 64 bits based on the given 114-bit keystream fragment. The PDSAT program was used to find partitionings with good time estimations for CNFs encoding this problem. The computational experiments were performed on the computing cluster “Academician V.M. Matrosov” of ISDCT SB RAS (http://hpc.icc.ru/index.php). One computing node of this cluster consists of 2 AMD Opteron 6276 CPUs (32 CPU cores in total). In each experiment PDSAT was launched for 1 day using 2 computing nodes (i.e. 64 CPU cores). We used random samples of size $N = 10^4$.

**Fig. 1:**
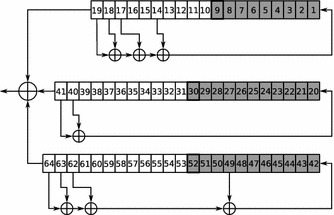
Decomposition set $S_1$ constructed in Semenov et al. (2011)

**Fig. 2:**
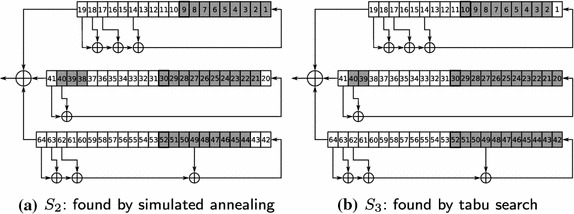
Decomposition sets found by PDSAT for cryptanalysis of A5/1. **a** $S_2$: found by simulated annealing. **b** $S_3$: found by tabu search

On Figs. 1, 2a, b three decomposition sets are shown. We described the first decomposition set (further referred to as $S_1$) in the paper Semenov et al. (2011). This set (consisting of 31 variables) was constructed “manually” based on the analysis of algorithmic features of the A5/1 generator. The second one ($S_2$), consisting of 31 variables, was found as a result of the minimization of $F\left( \cdot \right)$ by the simulated annealing algorithm (see “Algorithms for minimization of predictive function” section). The third decomposition set ($S_3$), consisting of 32 variables, was found as a result of minimization of $F\left( \cdot \right)$ by the tabu search algorithm. In the Table 1 the values of $F\left( \cdot \right)$ (in seconds) for all three decomposition sets are shown. Note that each of decomposition sets $S_2$ and $S_3$ was found for one 114 bit fragment of keystream that was generated according to the A5/1 algorithm for a randomly chosen 64-bit secret key.

### Solving cryptanalysis instances for A5/1 in the volunteer computing project SAT@home

The values of predictive function presented in Table 1 show that the SAT-based cryptanalysis of the A5/1 generator requires quite significant computing power. Specifically for the purpose of solving hard SAT instances we developed the volunteer computing project SAT@home (Posypkin et al. 2012). Volunteer computing (Durrani and Shamsi 2014) is a type of distributed computing which uses computational resources of PCs of private persons called volunteers. Each volunteer computing project is designed to solve one or several hard problems. SAT@home is based on the BOINC platform (Berkeley Open Infrastructure for Network Computing Anderson 2004). SAT@home was launched on September 29, 2011 by Matrosov Institute for System Dynamics and Control Theory of Siberian Branch of Russian Academy of Sciences and Kharkevich Institute for Information Transmission Problems of Russian Academy of Sciences. On February 7, 2012 SAT@home was added to the official list of BOINC projects (http://boinc.berkeley.edu/projects.php).

The experiment aimed at solving 10 cryptanalysis instances for the A5/1 keystream generator was held in SAT@home from December 2011 to May 2012. We used the rainbow-tables (http://opensource.srlabs.de/projects/a51-decrypt) to construct the corresponding instances. When analyzing 8 bursts of keystream (i.e. 912 bits) these tables allow to find the secret key with probability about 88%. We randomly generated 1000 instances and applied the rainbow-tables technique to analyze 8 bursts of keystream, generated by A5/1. Among these 1000 instances the rainbow-tables could not find the secret key for 125 problems. From these 125 instances we randomly chose 10 and in the computational experiments applied the SAT approach to the analysis of the first burst of each corresponding keystream fragment (114 bits). For each SAT instance we constructed the partitioning generated by the $S_1$ decomposition set (see Fig. 1) and processed it in the SAT@home project. All 10 instances constructed this way were successfully solved in SAT@home (i.e. we managed to find the corresponding secret keys) in about 5 months (the average performance of the project at that time was about 2 teraflops). The second experiment on the cryptanalysis of A5/1 was launched in SAT@home in May 2014. It was done with the purpose of testing the decomposition set found by tabu search algorithm. In particular we took the decomposition set $S_3$ (see Fig. 2b). On September 26, 2014 we successfully solved in SAT@home all 10 instances from the considered series.

It should be noted that in all the experiments the time required to solve the problem agrees with the predictive function value computed for the desomposition sets $S_1$ and $S_3$. Our computational experiments clearly demonstrate that the proposed method of automatic search for decomposition sets makes it possible to construct SAT partitionings with the properties close to that of “reference” partitionings, i.e. partitionings constructed based on the analysis of algorithmic features of the considered cryptographic functions.

### Time estimations for SAT-based cryptanalysis of Bivium and Grain

The Bivium keystream generator (Cannière 2006) is constructed from two shift registers of a special kind. The first one contains 93 cells and the second one contains 84 cells. The Grain keystream generator (Hell et al. 2007) also uses 2 shift registers: first is 80-bit nonlinear feedback shift register (NFSR), second is 80-bit linear feedback shift register (LFSR). To mix registers outputs the generator uses a special filter function *h*(*x*). In accordance with Maximov et al. (2007), Soos (2010) we considered cryptanalysis problems for Bivium and Grain in the following formulation. Based on the known fragment of keystream we search for the values of all registers cells at the end of the initialization phase. It means that we need to find 177 bits in case of Bivium and 160 bits in case of Grain.

Usually it is sufficient to consider keystream fragment of length comparable to the total length of shift registers to uniquely identify the secret key. Here we followed Eibach et al. (2008), Soos (2010) and set the keystream fragment length for Bivium cryptanalysis to 200 bits and for Grain cryptanalysis to 160 bits.

In our computational experiments we applied PDSAT to SAT instances that encode the cryptanalysis of Bivium and Grain according to the formulations described above. In these experiments to minimize the predictive functions we used only the tabu search algorithm, since compared to the simulated annealing it traverses more points of the search space per time unit. Also we noticed that the decomposition set for the A5/1 cryptanalysis, constructed by the tabu search algorithm, is closer to the “reference” set than that constructed with the help of simulated annealing.

**Fig. 3:**
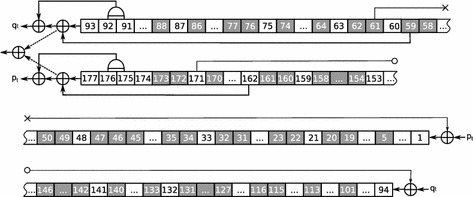
Decomposition set of 50 variables found by PDSAT for Bivium cryptanalysis

**Fig. 4:**
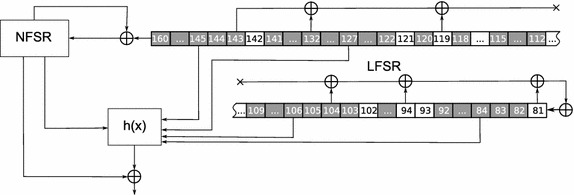
Decomposition set of 69 variables found by PDSAT for Grain cryptanalysis

During the cryptanalysis of Bivium and Grain in the role of $\tilde{X}_{start}$ we used the set formed by the variables encoding the cells of registers of the generator considered at the end of the initialization phase. Further we refer to these variables as *starting* variables. Thus $\left| \tilde{X}_{start}\right| =177$ in case of Bivium, and $\left| \tilde{X}_{start}\right| = 160$ in case of Grain. When computing predictive function values PDSAT used random samples of size $N=10^5$. It was launched for 1 day using 5 computing nodes (160 CPU cores in total) within the computing cluster “Academician V.M.Matrosov”. So there was 1 leader process and 159 computing processes. Time estimations obtained are $F_{best}=3.769\times 10^{10}$ for Bivium and $F_{best}=4.368\times 10^{20}$ seconds for Grain. Corresponding decomposition set $\tilde{X}_{best}$ for Bivium is marked with gray on Fig. 3 (50 variables) and the decomposition set for Grain is marked with gray on Fig. 4 (69 variables). Interesting fact is that $\tilde{X}_{best}$ for Grain contains only variables corresponding to the LFSR cells.

In Eibach et al. (2008), Soos et al. (2009), Soos (2010) a number of time estimations for SAT-based cryptanalysis of Bivium were proposed. In particular, in Eibach et al. (2008) several fixed types of decomposition sets (*strategies* in the notation of Eibach et al. (2008)) were analyzed. The best decomposition set from Eibach et al. (2008) consists of 45 variables encoding the last 45 cells of the second shift register. Note that in Eibach et al. (2008) the corresponding estimation of time equal to $1.637\times 10^{13}$ was calculated using random samples of size $10^2$. In Soos et al. (2009), Soos (2010) the estimations of runtime for CryptoMiniSat solver, working with SAT instances encoding Bivium cryptanalysis, were presented. From the description of experiments in these papers it can be seen that authors used probabilistic experiment to estimate the sets of variables chosen by CryptoMiniSat during the solving process and extrapolated the estimations obtained to time points of the solving process that lay in the distant future. Note that in Soos et al. (2009), Soos (2010) the problem of estimating the effectiveness of a particular partitioning is not considered as the problem of estimating the expected value of some random variable (that is necessary for it to correspond to the Monte Carlo method in its classical sense). Apparently, as it is described in Soos et al. (2009), Soos (2010), the random samples of size $10^2$ and $10^3$ were used. In the Table 2 all three estimations mentioned above are shown. The performance of one CPU core we used in our experiments is comparable with that of one CPU core used in Soos et al. (2009), Soos (2010).

### Solving cryptanalysis instances for Bivium and Grain

Since the values of predictive functions for Bivium and Grain cryptanalysis turned out to be quite large, in our computational experiments we studied “weakened” variants of the corresponding instances. For this purpose we used the sets of the so called “guessing bits” (Bard 2009). The instances obtained were solved on a computing cluster (with the help of PDSAT) and in the SAT@home project.

In the solving mode of PDSAT for $\tilde{X}_{best}$ found during predictive function minimization all $2^{\left| \tilde{X}_{best}\right| }$ assignments of variables from $\tilde{X}_{best}$ are generated. PDSAT solves all corresponding SAT instances. To compare obtained time estimations with real solving time we used PDSAT to solve several cryptanalysis problems for Bivium and Grain with several known guessing bits. Below we use the notation *BiviumK* (*GrainK*) to denote the cryptanalysis of Bivium (Grain) with known *K* guessing bits. In the role of guessing bits in all cases we chose known values of *K* starting variables encoding the last *K* cells of the second shift register. We solved 3 instances for each of the following problems: Bivium16, Bivium14, Bivium12, Grain44, Grain42 and Grain40.

In the following experiments for each BiviumK (GrainK) problem we computed the estimation for the first instance from the corresponding series and used the obtained decomposition set for all 3 instances from the series. To get more statistical data we did not stop the solving process after the satisfying assignment was found, thus processing the whole decomposition family. In the Table 3 for each problem we show the time required to solve it using 15 computing nodes (480 CPU cores total) of “Academician V. M. Matrosov”. The estimation of time was computed for the first instance (inst. 1) in all cases. The estimation for 480 CPU cores is based on the estimation for 1 CPU core. According to the results from this table, on average the real solving time deviates from the estimation by about 8 %.

We also solved the Bivium9 problem in SAT@home. With the help of PDSAT the decomposition set formed of 43 variables was found. Using this decomposition set 5 instances of Bivium9 were solved in SAT@home in about 4 months from September 2014 to December 2014. During this experiment the average performance of the project was about 4 teraflops.

It should be noted that for all considered BiviumK and GrainK problems the time required to solve the corresponding instances on the computing cluster and in SAT@home agrees well with values of the predictive function found by our approach.

## Related work

Apparently, the paper Cook and Mitchell (1997) was the first work in which it was proposed to use SAT encodings of inversion problems of cryptographic functions as justified hard SAT instances. One of the first examples of SAT encodings for a widely known ciphering algorithm was proposed in Massacci and Marraro (2000): in particular, in that paper the process of constructing SAT encoding for the DES algorithm was described. To the best of our knowledge, the first example of successful application of SAT solvers to cryptanalysis of real-world cryptographic functions was given in Mironov and Zhang (2006). It used the SAT solvers to construct collisions for the hash functions from the MD family.

The monograph (Bard 2009) contains systematic research of various questions regarding algebraic cryptanalysis. A substantial part of this book studies the possibilities of the use of SAT solvers for solving cryptanalysis equations represented in the form of algebraic systems over finite fields.

The A5/1 algorithm is still used in many countries to cipher GSM traffic. During the long lifetime of this algorithm a lot of attacks on it have been created. However, the first attacks that allowed to find the secret key in manageable time were presented by the A5/1 Cracking Project Group in 2009 [27]. These attacks were in fact developed from the Rainbow method (Oechslin 2003). In Güneysu et al. (2008) a number of techniques, used in the A5/1 Cracking Project to construct Rainbow tables, were presented. The cryptanalysis of A5/1 via Rainbow tables has the success rate of approximately 88 % if one uses 8 bursts of keystream. The success rate of the Rainbow method if one has only 1 burst of keystream is about 24 %. In all our computational experiments we analyzed the keystream fragment of size 114 bits, i.e. one burst, and considered only instances for which the solution could not be found using the Rainbow method. We successfully solved in SAT@home several dozens of such instances. In Semenov et al. (2011) we described our first experience on the application of the SAT approach to A5/1 cryptanalysis in the specially constructed grid system BNB-Grid. In that paper we found the set $S_1$ (see “Time estimations for SAT-based cryptanalysis of A5/1” section) manually, based on the peculiarities of the A5/1 algorithm.

The Bivium generator is a weakened variant of the Trivium generator (Cannière 2006) developed within the context of the eSTREAM project. The detailed analysis of its vulnerabilities was performed in Maximov et al. (2007). As far as we know, the cryptanalysis estimations from that paper were not verified with the exception of the distinguishing attack. Later the Bivium generator became quite a popular object of the SAT-based cryptanalysis. The paper Mcdonald et al. (2007) was the first research in that direction. In Eibach et al. (2008) there was described the SAT-based attack on Bivium, which used specially constructed sets of guessing bits. One of the advantages of Eibach et al. (2008) consists in the fact that their computational experiments are easy to reproduce. In Soos et al. (2009), Soos (2010) there was constructed a time estimation for the SAT-based cryptanalysis of Bivium, that was much better than all previous estimations. Essentially, to construct it the Monte Carlo method was used (in Soos 2010 the author even uses the term “Monte Carlo algorithm”). However that paper does not really contain any references to theoretical basics of the method: there is no formal definition of the random variable, the expected value of which is estimated. The main novelty of our approach consists in strict justification of the applicability of the Monte Carlo method to estimating the effectiveness of SAT partitionings, and in using metaheuristic algorithms (simulated annealing and tabu search) for finding partitionings with good estimations of total time required to process them.

The Monte Carlo method for estimating the expected value of a random variable was first proposed in Metropolis and Ulam (1949). There are a lot of modern guides and handbooks containing the description and the results of application of this method, for example, Kalos and Whitlock (1986).

Simulated annealling was first described in Kirkpatrick et al. (1983). It is used to solve optimization problems from various areas. Tabu search is another widely used metaheuristic method originated from Glover and Laguna (1997).

The questions regarding solving SAT in parallel and distributed environments were considered in a number of papers. In particular, in Hyvärinen (2011) a systematic review of methods for constructing SAT partitionings is presented.

The grid systems aimed at solving SAT are relatively rare. In Schulz and Blochinger (2010) a desktop grid for solving SAT which used conflict clauses exchange via a peer-to-peer protocol was described. Apparently, Black and Bard (2011) became the first paper about the use of a desktop grid based on the BOINC platform for solving SAT. Unfortunately, it did not evolve into a full-fledged volunteer computing project. The predecessor of the SAT@home was the BNB-Grid system (Evtushenko et al. 2009; Semenov et al. 2011), that was used to solve first large scale SAT-based cryptanalysis problems in 2009.

At the present moment there are several common principles that lie in the basis of modern SAT solvers. From many years of our experience we believe that in application to cryptanalysis instances the best solvers are the ones based on the CDCL concept (Marques-Silva et al. 2009). It might seem surprising that CDCL solvers show good results even when we solve inversion problems for functions with large number of preimages (for example, when we search for collisions of cryptographic hash functions). Nowadays there are many CDCL-solvers that have a common basic architecture but differ in details and heuristics.

## Conclusion

In the present paper we propose the method for constructing SAT partitionings for solving hard SAT instances in parallel. This approach is based on the Monte Carlo method (in its classical form) for estimating expected value of random variable. From our point of view the proposed method and the corresponding algorithms can be used in SAT-based cryptanalysis, that is an actively developing direction in cryptography. We tested our method in application to cryptanalysis of several keystream generators (A5/1, Bivium, Grain). In the nearest future we are going to expand the list of metaheuristics used for minimization of predictive functions. Also we plan to investigate the question of accuracy of the estimations obtained by the Monte Carlo method for the considered class of problems in more detail.

**Table 1:** Decomposition sets for SAT-based cryptanalysis of A5/1 and corresponding values of the predictive function

Set	Power of set	$F\left( \cdot \right)$
$S_1$	31	4.45140e+08
$S_2$	31	4.78318e+08
$S_3$	32	4.64428e+08

**Table 2:** Time estimations for the Bivium cryptanalysis problem

Source	*N*	Time estimation
From Eibach et al. (2008)	$10^{2}$	$1.637\times 10^{13}$
From Soos et al. (2009), Soos (2010)	$10^{3}$	$9.718\times 10^{10}$
Found by PDSAT	$10^{5}$	$3.769\times 10^{10}$

**Table 3:** Solving cryptanalysis problems for Bivium and Grain on a computing cluster

Problem	$\left| \tilde{X}_{best}\right|$	$F_{best}$		$\Delta _C(\tilde{X}_{best})$ on 480 cores			Finding SAT on 480 cores		
		1 core	480 cores	inst. 1	inst. 2	inst. 3	inst. 1	inst. 2	inst. 3
Bivium16	31	1.65e7	3.44e4	3.42e4	3.36e4	3.42e4	1.10e3	2.33e4	2.67e4
Bivium14	35	6.84e7	1.42e4	1.34e5	1.32e5	1.33e5	3.95e2	9.10e4	9.18e4
Bivium12	37	2.63e8	5.50e5	4.95e5	4.83e5	5.28e5	3.04e5	1.39e5	1.89e5
Grain44	29	1.60e7	3.36e4	3.61e4	4.51e4	3.73e4	1.34e3	1.35e4	8.24e2
Grain42	29	6.05e7	1.26e5	1.35e5	1.30e5	1.20e5	6.92e4	1.07e5	9.15e4
Grain40	32	2.52e8	5.27e5	5.79e5	5.73e5	5.06e5	3.10e5	5.10e5	3.20e5
